# The Cardiac Arrest Support Tier: a service evaluation

**DOI:** 10.29045/14784726.2020.09.5.2.38

**Published:** 2020-09-01

**Authors:** Matthew Metcalf, Matthew Robinson, Pippa Hall, James Goss

**Affiliations:** South Western Ambulance Service Foundation Trust Hazardous Area Response Team; Great Western Air Ambulance Charity; South Western Ambulance Service Foundation Trust Hazardous Area Response Team; South Western Ambulance Service Foundation Trust Hazardous Area Response Team

**Keywords:** paramedic, pre-hospital, resuscitation

## Abstract

**Aim:**

This service evaluation seeks to determine whether the pre-hospital Cardiac Arrest Support Tier (CAST), implemented by a Hazardous Area Response Team (HART), was clinically effective, feasible and acceptable during its pilot year.

**Methods:**

Chest compression feedback, provision of Return of Spontaneous Circulation (ROSC) care and CAST paramedic exposure to Out-of-Hospital Cardiac Arrest (OHCA) were audited. The number of incidents that CAST responded to and the number of staff it committed were also assessed. An online questionnaire was used to gauge acceptability of the project among frontline Ambulance Service Trust staff.

**Results:**

CAST attended 178 OHCAs and committed a median of three (IQR 2–3) paramedics to each incident. In comparison to data from both South Western Ambulance Service Foundation Trust (SWASFT) and the National Ambulance Service in England, CAST delivered the full complement of post-ROSC care more frequently during the same period (CAST = 80% vs SWASFT = 68.5% vs England = 77.46%). CAST paramedics had a median exposure to 15.5 (IQR 12–19) OHCAs during the pilot year. Unfortunately, chest compression feedback was unavailable due to ongoing equipment inaccuracies and failure.

Additionally 64.6% (n = 42/65) of SWASFT respondents believed CAST to be beneficial to resuscitation attempts, 63.1% (n = 41/65) would like CAST to continue to support resuscitation attempts in the future and 55.6% (n = 35/63) felt supported by CAST staff on scene.

**Conclusion:**

CAST is logistically feasible, is acceptable to the majority of SWASFT staff and demonstrated the successful delivery of evidence-based practice (EBP) to OHCA incidents.

## Introduction

In England in 2017, Emergency Medical Services (EMS) attempted resuscitation on approximately 32,000 patients who suffered an Out-of-Hospital Cardiac Arrest (OHCA) (NHS England, 2020). With a national average survival rate of less than 9%, the United Kingdom (UK) compares poorly to other developed countries (Wissenburg et al., 2013). With similar survival rates seen locally, the Medical Directorate of the South Western Ambulance Service Foundation Trust (SWASFT) launched their ‘Saving Lives Together’ campaign (SWASFT, 2018a). Currently only in its preliminary stages, the strategy looks for initiatives to help improve the Trust’s provision of cardiac arrest care.

UK paramedic-led, specialist resources for OHCA have previously reported improved patient outcomes (Clarke et al., 2014; McClelland et al., 2016). With scope to dedicate a specific resource to respond to OHCAs, in concurrence with existing responsibilities (Phillips, 2017), SWASFT Hazardous Area Response Team (HART) paramedics implemented a secondary response tier to attend and support resuscitation attempts in the Greater Bristol area.

This work aims to evaluate the Cardiac Arrest Support Tier’s (CAST) pilot year to determine whether the response model is clinically effective, feasible and acceptable to SWASFT staff. If so, it may have the potential to become a standard response model for the area or to be adopted in other localities.

## Methods

### Background

HART comprises paramedics who are specially trained to deliver clinical care to patients in hazardous environments. It exists across all Ambulance NHS Trusts in England, with SWASFT bases located in Bristol and Exeter (Metcalf, 2018). A team of six HART paramedics provide 24-hour cover from their base of operations. Staffing typically consists of a Team Leader, a Lead Paramedic and four Operatives, working on two double-crewed vehicles and two single-crewed vehicles to respond to incidents across Bristol, Somerset, Gloucestershire and Wiltshire.

HART paramedics from Bristol, inspired by previous UK pre-hospital cardiac arrest initiatives (Clarke et al., 2014; McClelland et al., 2016), proposed implementation of a designated cardiac arrest team. CAST aimed to respond to all OHCAs and to support frontline ambulance staff in providing high-quality pre-hospital resuscitation.

The SWASFT Resuscitation Group approved the launch of a pilot year in the Greater Bristol area. Subsequently, 22 eligible HART paramedics volunteered to undertake additional training of technical and non-technical skills in cardiac arrest management, facilitated and assessed by the Trust’s Medical Director for Acute Care. All candidates had received prior enhanced care skills training and assessment, including surgical airway and finger thoracostomy. Additional training on an automated chest compression device was also provided.

The team had direct access to a 24-hour advice line staffed by a Consultant in Pre-Hospital Critical Care, who could support interventions and decision-making. On 3 September 2018, the CAST pilot began operating in Bristol.

The Team Leader was notified by the HART dispatcher of all patients in cardio-respiratory arrest or ROSC within an approximate 20-minute drive from base. Operatives were dispatched on a case-by-case basis with at least two responding CAST-trained clinicians. HART incidents took precedence over CAST response.

### Measures

The study of CAST’s pilot year will be carried out as a service evaluation (SE) with certain clinical aspects, where best practice standards exist, taking the form of an audit. This work will be presented using the revised Standards for Quality Improvement Reporting Excellence (SQUIRE) 2.0 guideline.

#### Clinical effectiveness

ROSC and survival to hospital discharge are important and widely used measures of outcome in cardiac arrest studies (Schluep et al., 2018). These have been used by previous SEs of other UK pre-hospital cardiac arrest teams (Clarke et al., 2014; McClelland et al., 2016; Pilbery et al., 2019). These studies, however, are limited by confounding variables and bias. This results in poor internal validity, and consequently causal relationships between variables, such as cardiac arrest team attendance and patient outcomes, cannot be reliably evidenced (Sackett et al., 1996). This could only be achieved with high-quality experimental or potentially prospective observational research (Song & Chung, 2010). Implying that these teams improve patient outcomes in the absence of a research design is likely to be misleading.

The investigators of this SE lacked the time, resources and expertise to conduct a research study. Nonetheless, there was still the requirement to formally evaluate the implementation of a new service to ensure that it delivers the expected level of care (Moule et al., 2016). Therefore, this SE required an alternative and novel method of assessing the clinical effectiveness of CAST. It is well documented that high-quality cardiopulmonary resuscitation (CPR) improves patient outcomes (Christenson et al., 2009; Edelson et al., 2006; Idris et al., 2012; Kirves et al., 2007; Ko et al., 2005; Kramer-Johansen et al., 2006; Steen & Kramer-Johansen, 2008). In the absence of patient-focused outcomes, measuring CPR quality can provide quantitative data on the provision of an evidence-based practice (EBP) known to influence outcomes. CAST uses CPR feedback devices at OHCA, whereas the rest of SWASFT (2018b) routinely does not, so a comparison between services cannot be drawn. Therefore, CPR feedback data on depth, frequency and compression fraction for each resuscitation that CAST supported was audited against the Resuscitation Council UK (RCUK) (2015) standards, as summarised in Table 1. The percentage of incidents where the means for all aspects of CPR meet the standard will determine with what frequency clinically effective care is delivered.

**Table 1.** RCUK (2015) CPR standards.

CPR aspect	RCUK (2015) standard
Chest compression frequency	100–120 compressions per minute
Chest compression fraction	< 10 seconds
Chest compression depth	3–5 cm

Post-resuscitation care is the last link in the chain of survival (Quinn et al., 2017). Protocol-driven care must be delivered consistently to improve outcomes (Knafelj et al., 2007; Oddo et al., 2006; Sunde et al., 2007). Through the provision of a care bundle, pre-hospital responders can influence post-ROSC patient outcomes (Page, 2012). Consequently, NHS England (2020) has developed a post-ROSC care bundle (Table 2) that NHS Ambulance Service Trusts have compliance audited against quarterly. 100% of the care bundle must be delivered to be compliant unless an exclusion criterion is met. It was deemed appropriate by the investigators of this SE to evidence clinical effectiveness further by auditing CAST’s provision of the full post-ROSC care bundle and comparing the results to the rest of SWASFT and the ambulance service in England for the same year.

**Table 2.** NHS England (2020) post-resuscitation care bundle.

Component of post-ROSC care bundle	Exceptions
12-lead ECG taken post ROSC	Patient refusal Patient re-arrested with ROSC < 10 minutes in duration
Blood glucose recorded post ROSC	Patient refusal Patient re-arrested with ROSC < 10 minutes in duration Blood glucose measured prior to ROSC and within normal range
End-tidal CO_2_ reading/waveform recorded post ROSC / continuously	Patient refusal Patient re-arrested with ROSC < 10 minutes in duration Not required: no advanced airway in situ
Oxygen administered post ROSC / continuously	Patient refusal Patient re-arrested with ROSC < 10 minutes in duration Not required: oxygen saturations were 94–98% (88–92% if chronic obstructive pulmonary disease)
Systolic blood pressure reading recorded post ROSC or, if unobtainable, presence of radial pulse documented	Patient refusal Patient re-arrested with ROSC < 10 minutes in duration
Administration started of a 250 ml bolus of saline fluids post ROSC	Patient refusal Patient re-arrested with ROSC < 10 minutes in duration Not required: systolic blood pressure > 90 or presence of radial pulse where blood pressure is unobtainable, evidence of significant heart failure or hypervolemia clearly documented All attempts to gain intravenous and intraosseous vascular access are unsuccessful
*Exclude Traumatic Cardiac Arrest, patients successfully resuscitated before the arrival of ambulance staff, ROSC achieved en route or upon arrival at hospital and patients aged less than 18 years*	

Recent research suggests that paramedics with a greater frequency of exposure to OHCA are associated with better patient outcomes (Dyson et al., 2016; Tuttle & Hubble, 2018). Both studies were unable to explain the specific resuscitation traits that are responsible for this but hypothesise that responders with the most experience are able to more frequently practise resuscitation skills and maintain competency, factors known to improve CPR quality (Soar et al., 2010). It is expected that individual CAST paramedic exposure to OHCA will be greater than that of regular SWASFT paramedics. To show that the benefits of this evidence-based relationship are being delivered to OHCA incidents in the Bristol area, CAST paramedic frequency of exposure to OHCA was measured and compared to figures from the studies of Tuttle and Hubble (2018) and Dyson et al. (2016).

#### Feasibility

The CAST service evaluation examined how many OHCAs were responded to by CAST and the number of CAST paramedics committed to each OHCA attended.

#### Acceptability

An optional, internet-based, anonymised, self-completion, closed-ended questionnaire was emailed to Trust staff at four local ambulance stations within the CAST response area. This was done to ascertain the acceptability of the service among SWASFT staff. Unfortunately, it was beyond the capabilities of the investigators to analyse rich qualitative data, so free text boxes were not included.

### Data collection

Anonymised data were collected from CAST paramedic self-report forms, electronic patient care records (ePCRs), the SWASFT Special Operations log, SWASFT incident Sequence of Events logs and CAST’s monitoring and defibrillation equipment. Data not available to the investigator were requested from the SWASFT Research Audit and Quality Improvement Department and Clinical Information and Records Office.

## Results

### Patient demographics

**Table 3.** Patient demographics.

Demographic	Frequency
Number	178
Age median (IQR) years	62 (45–76)
Male n (%)	114 (64%)
Bystander CPR n (%)	129 (72.5%)
Shockable rhythm n (%)	42 (23.6%)
Non-shockable rhythm n (%)	136 (76.4%)
Presumed traumatic cause n (%)	24 (13.5%)
Presumed medical cause n (%)	154 (86.5%)

### Chest compression feedback

After repeated reports of absent or inaccurate CPR feedback at OHCAs, the CPR feedback device was withdrawn from use for the remainder of the pilot year.

### Post-ROSC care bundle provision

**Figure 1:**
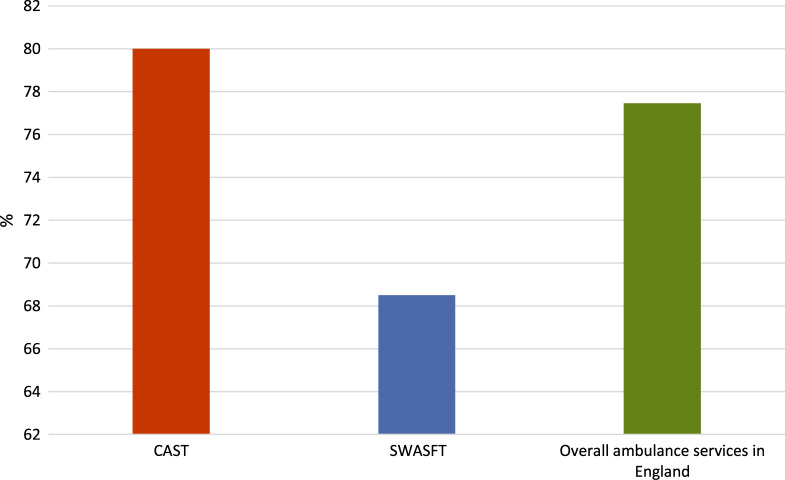
Figure 1. Percentages of incidents where the full national post-ROSC care bundle was completed for CAST, SWASFT and other ambulance services in England.

CAST supported the management of 58 ROSC patients in the pilot year. Of these, 20 were managed without a senior clinical asset leading, such as the local Critical Care Team or BASICS Doctor. For clarity, only these 20 incidents where CAST supported Trust resources independently will be analysed. 80% (n = 16) had the complete post-ROSC care bundle delivered, a rate greater than those of SWASFT and the ambulance service in England for the same period (SWASFT = 68.5%, England = 77.46%) (Figure 1).

### CAST paramedic individual exposure to OHCA

CAST paramedics had a median exposure to 15.5 (IQR 12–19) OHCAs in the pilot year. This volume of exposure is greater than those found to improve patient outcomes by the evidence base (≥ 15 OHCA in 5 years improves chances of ROSC (Tuttle & Hubble, 2018) and ≥ 6 OHCA in 3 years improves chances of survival to hospital discharge (Dyson et al., 2016)).

### CAST frequency of response and resourcing

**Figure 2:**
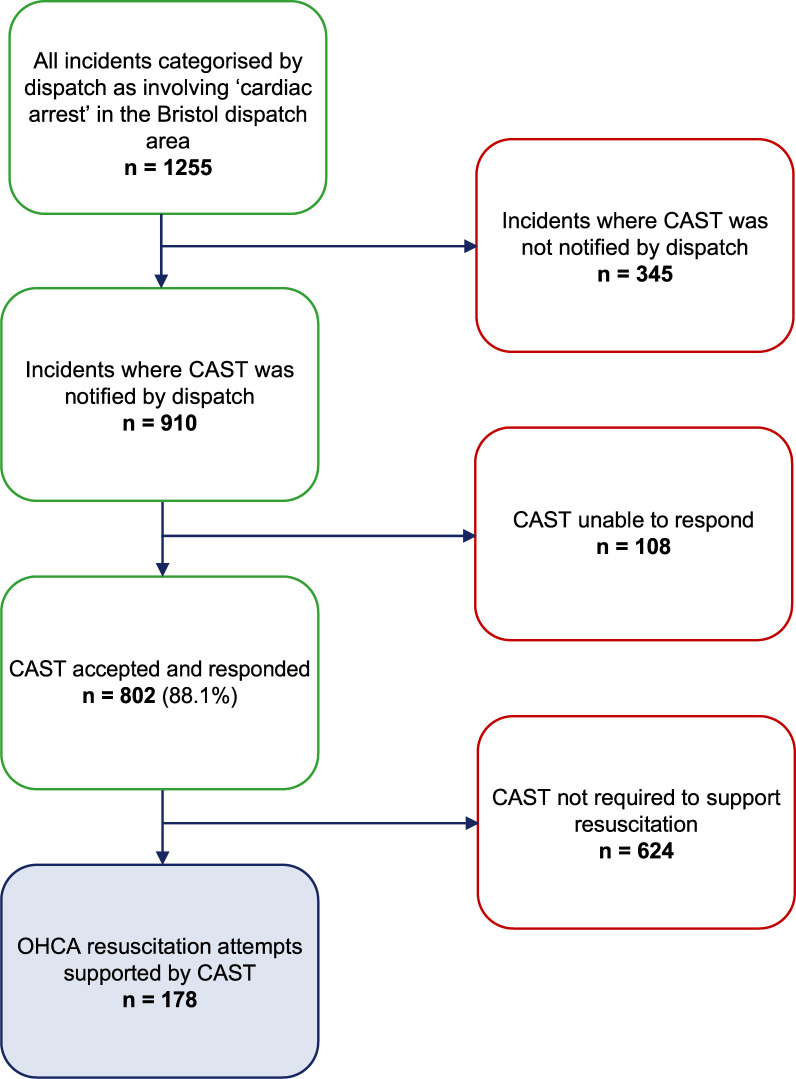
Figure 2. Patient selection flow diagram.

CAST responded to 802 (88.1%) of the 910 OHCAs that dispatchers notified them of (Figure 2) without compromising normal service delivery. This resulted in 178 OHCA resuscitation attempts being supported. CAST was stood down on 624 incidents for reasons such as the incident being not as given or patient not for resuscitation. CAST committed a median of three (IQR 2–3) paramedics to scene at each resuscitation it supported, and provided a total of 551 members of staff to support OHCA in the pilot year.

### Questionnaire

64.6% (n = 42/65) of respondents felt that CAST was beneficial to resuscitation attempts, 63.1% (n = 41/65) would like CAST to continue to support resuscitation attempts in the future and 55.6% (n = 35/63) felt supported by CAST staff when they attended the scene (Table 4).

**Table 4.** Self-complete questionnaire answers.

Question	Answered yes
From your own experiences, do you think CAST is beneficial to resuscitation attempts?	42/65 (64.6%)
Would you like CAST to continue to support resuscitation attempts?	41/65 (63.1%)
Do you feel supported by CAST staff when they attend scene?	35/63 (55.6%)
Are you aware of the CAST CPD event run by the Hazardous Area Response Team?	29/65 (44.6%)
Would you like to attend a CAST-run CPD event in the future?	50/65 (76.9%)

## Discussion

### Feasibility

CAST appears to be only the fourth specialist, pre-hospital cardiac arrest team to operate in the UK after those reported by Clarke et al. (2014), McClelland et al. (2016) and Pilbery et al. (2019). More significantly though, SWASFT HART Bristol is the first HART unit in the UK to provide a service of this type. In its pilot year, this novel response model was able to respond to the vast majority of OHCA incidents it was notified of, and demonstrated that it is feasible for HART Bristol to sustain operating CAST. CAST’s frequency of attendance to OHCA was greater than that of the pilot years of other UK pre-hospital cardiac arrest teams of comparable design (Phillips, 2017), and accounted for one in 20 of SWASFT’s total resuscitation attempts for the same period. The cost of this service is absorbed entirely by the HART Bristol budget with no significant financial impact. The potential benefits of a response model of this type could be extended further with the implementation of additional cardiac arrest teams at HART Exeter or other HART units nationally, or by using models independent of HART elsewhere in the Trust.

Further feasibility was demonstrated as CAST responded approximately three members of staff to every OHCA it supported in the pilot year with no reported compromise to HART’s normal operational commitments. These additional resources meant theoretically fewer Trust resources were required and could therefore help relieve pressures currently being experienced elsewhere in the ambulance service (Quaile, 2016). Unfortunately, this was not the case, as CAST’s resources, in addition to an unreduced standardised SWASFT response, often led to significant personnel on scene. The impact of this, however, is difficult to quantify as the relationship between cardiac arrest team size and cardiopulmonary resuscitation quality is poorly understood in the literature (Hunziker et al., 2018). Nevertheless, work must be carried out with dispatchers to optimise and then formalise a local response model to reduce over-resourcing. This should be done with consideration for emerging research concerning the future of other pre-hospital support assets’ OHCA response (von Vopelius-Feldt et al., 2019).

### Clinical effectiveness

The decision to remove CAST’s chest compression feedback device from service was one that significantly impacted this SE’s ability to measure the quality of clinical care provided. This decision was, however, appropriate and unavoidable in the interests of reliability and patient safety. At the time of publication, the manufacturers of this device are aware of these issues and are working to resolve them. No other SWASFT resource uses the same device. It is not known whether the device is in service with other ambulance services in the UK. In the absence of chest compression feedback data, greater importance was placed on other clinical measures to determine clinical effectiveness.

**Figure 3:**
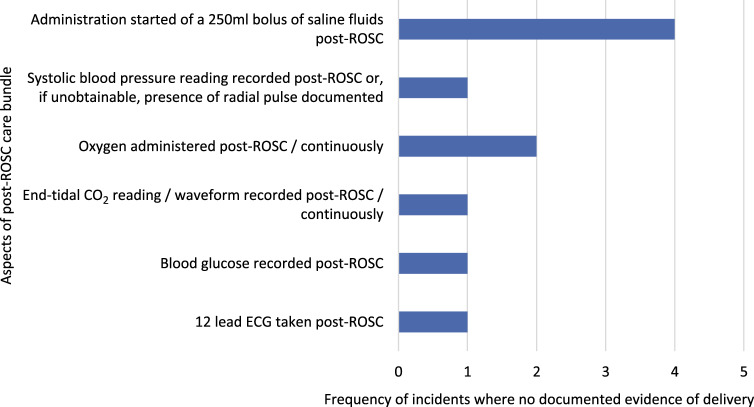
Figure 3. The aspects and frequencies of the post-ROSC care bundle not delivered on the four ROSC incidents where the full post-ROSC bundle was not achieved by CAST.

CAST paramedics demonstrated high rates of evidence-based post-resuscitation care delivery, which was provided more frequently than the rest of the Trust and ambulance services nationally. Figure 3 highlights which aspects of the bundle were not delivered by clinicians at each of these incidents. All aspects of this bundle are basic interventions that paramedics are familiar with through the Joint Royal Colleges Ambulance Liaison Committee (JRCALC, 2019) guidelines. Their omission by paramedics is therefore surprising. Recordkeeping at OHCA is frequently carried out retrospectively and information is often inputted by any member of ambulance staff present. Research by Ho et al. (2017) demonstrated that EMS clinical documentation from memory is significantly inaccurate. Memory is known to be imprecise, especially when under stress (Sandi, 2013). This could explain the absence of documentation of these interventions. If so, CAST paramedics must ensure future records are accurately documented and handed over (Mann & Williams, 2003). Failure to do so is potentially in breach of the Health and Care Professions Council’s (2016) Standards of Conduct, Performance and Ethics.

The authors of this work have decided not to interpret CAST patient outcomes due to the limitations of the study design. For transparency, however, overall and Utstein outcomes are reported in Table 5 but will not be compared to other services. The Utstein subgroup are patients who have an initial shockable rhythm and bystander CPR, known predictors for survival (Perkins et al., 2015).

**Table 5.** Patient outcomes for CAST.

	Overall		Utstein group	
	ROSC	Survival to hospital discharge	ROSC	Survival to hospital discharge
**CAST**	32.6%	7.3%	62.5%	34.4%

Dyson et al. (2016) argue that paramedics with the highest exposure to OHCA should act as cardiac arrest specialists in a team. CAST paramedic individual exposure to OHCA was greater than those found to improve patient outcomes in the literature (Dyson et al., 2016; Tuttle & Hubble, 2018). In the absence of reliable data, this measure serves to reassure us that if a case–volume relationship does indeed exist, CAST paramedic attendance to OHCA will consistently deliver the benefits of that relationship to scene.

Caution is required when exposing CAST paramedics to a higher frequency of OHCAs, as exposure to traumatic events can lead to an increased risk of Post-Traumatic Stress Syndrome in ambulance service staff (Bennett et al., 2005). Regular undertaking of the family liaison role and communicating bad news has the potential to be detrimental to CAST paramedics. This issue among clinicians in general is reflected by the work of Smith-Cumberland & Feldman (2006). The beneficial clinical implications for the patient of greater paramedic exposure to OHCA must be balanced with the potential detrimental impact on responders. On the other hand, however, and as reported by CAST paramedics, regular exposure to these incidents can also lead to personal growth and a greater sense of self-efficacy (Blackburn & Owens, 2014; Linley & Joseph, 2004; Nygaard et al., 2016; Roditi et al., 2019).

### Acceptability

CAST provides clinical decision-making, specialist equipment and experienced guidance on the patient’s clinical course. For example, Trust staff were supported to end protracted resuscitation attempts outside of normal guidance in approximately a third of CAST’s attendances, utilising the consultant-led advice line. This not only enabled ambulance staff to feel supported in stressful incidents, but also expedited the process of a dignified death for the patient in cases of futility.

The impact of this support appears to be reflected in the results of the CAST questionairre. For the majority of SWASFT staff in Bristol who responded to the questionnaire, CAST is felt to be beneficial to resuscitation attempts, supportive of staff and something they would like to see continue to support resuscitation attempts in the future. Although this is encouraging, work needs to be carried out to understand the specific reasons for the negative responses. Qualitative data may have achieved this.

CAST provided outreach visits to hospitals and ambulance stations to engage with SWASFT staff and make them aware of the imminent launch of the pilot project. Furthermore, two Continuing Professional Development (CPD) events were run for all grades of frontline Trust staff and Community First Responders, to highlight the principles of CAST and run simulated resuscitation scenarios and skill stations. The CAST questionnaire revealed that approximately three quarters of respondents would like to attend this event in the future.

### Limitations

This work was conducted as an SE, and so conclusions are not generalisable or transferable. Furthermore, all data are subject to bias and confounding variables inherent with non-research study designs.

This study is limited by its inability to evidence CAST’s impact on outcomes. This is compounded by the loss of chest compression feedback due to technical issues.

CAST paramedic individual exposure to OHCA was recorded using self-report. This was unfortunately the only method available for the investigators to collect data on exposure. This method has the potential to result in missing data and thus selection bias (Rothman et al., 2008).

A closed-end design questionnaire was used as it offered a low-cost, far-reaching tool that required little specialist equipment (Boynton & Greenhalgh, 2004). This method offered only a low depth of answer and potentially introduced bias to responses.

Finally, ePCR data were collected for several measures. This data can be inputted by any staff member on scene at an OHCA incident and may be unintentionally inaccurate and thus unreliable.

## Conclusion

This novel pre-hospital cardiac arrest team is feasible for both HART and Ambulance Trusts, evidenced by the response to a high number of OHCAs without compromise to HART’s primary operational responsibilities.

CAST paramedics have a significantly increased exposure to OHCA, which has potential to improve patient outcomes at pre-hospital resuscitation attempts. However, this must be balanced with the potential detriment to a responder’s welfare.

CAST has demonstrated an improved post-ROSC care bundle in comparison to Trust and national figures. In the absence of outcome measures, these comparisons offer insight into the EBP to which CAST has aligned. It is unfortunate that chest compression feedback could not be used as a measurable outcome due to equipment malfunction.

If utilised properly, this service model could be developed by ambulance services across the UK to help reduce pressures on service delivery and to offer more OHCA patients access to a service with potential clinical benefit. CAST must now work with dispatchers to optimise and formalise a local response model to enhance cardiac arrest care and reduce over-resourcing.

The majority of questionnaire respondents felt that CAST was an acceptable and supportive model. Nonetheless, further work needs to be undertaken to understand the perceptions of staff who do not share this view.

## Acknowledgements

The authors would like to thank Kerron Allen, Dr Sarah Black, Paul Brett, Dr Philip Cowburn, Raul Hall, Rhys Hancock, Lucy Hayward, Christopher Hewett, Martyn Hill-Whatmore, Loraine Howells, Greg Leeson, Jessica Lynde, Katherine McNee, Edward Mellor, Anna Metcalf, Arthur Metcalf, Frederick Metcalf, Stephanie Moreland, Steven Pawley, Christopher Read, Peter Sadler, Amy Sainsbury, Matthew Turnock, Andrew Weston and Christian Wiggin, and to acknowledge the support, dedication and professionalism of the HART Bristol CAST paramedics.

## Author contributions

MM was involved in setting up and developing CAST; designed and led this SE; collected, analysed and interpreted data; and wrote and revised the article. MR designed CAST and led its set-up and development; and had input into writing and reviewing the manuscript. PH was involved in setting up and developing CAST; collected, analysed and interpreted data; and had input into writing and reviewing the manuscript. JG was involved in setting up and developing CAST; collected, analysed and interpreted data; and had input into writing and the reviewing manuscript. MM acts as the guarantor for this article.

## Conflict of interest

All authors of this work led or developed the CAST project.

## Ethics

No NHS Research Ethics Committee (REC) approval was required for this service evaluation. Instead, the SWASFT non-research project proposal process was followed, with approval being received from SWASFT RAQID on 26 November 2018. Ethical considerations for SEs, outlined by the Healthcare Quality Improvement Partnership (2011), were followed and a project risk assessment was carried out.

## Funding

None.
